# The management of Asherman syndrome: a review of literature

**DOI:** 10.1186/1477-7827-11-118

**Published:** 2013-12-27

**Authors:** Alessandro Conforti, Carlo Alviggi, Antonio Mollo, Giuseppe De Placido, Adam Magos

**Affiliations:** 1 University Department of Obstetrics, Gynaecology, Urology and Reproductive Medicine, University of Naples Federico II, Via Sergio Pansini n. 6, Naples 80100, Italy; 2 University Department of Obstetrics and Gynaecology, Royal Free Hospital, London NW3 2QG, UK

**Keywords:** Asherman syndrome, Intrauterine adhesion, Hysteroscopy, Endometrial regeneration

## Abstract

Asherman syndrome is a debatable topic in gynaecological field and there is no clear consensus about management and treatment. It is characterized by variable scarring inside the uterine cavity and it is also cause of menstrual disturbances, infertility and placental abnormalities. The advent of hysteroscopy has revolutionized its diagnosis and management and is therefore considered the most valuable tool in diagnosis and management. The aim of this review is to explore the most recent evidence related to this condition with regards to aetiology, diagnosis management and follow up strategies.

## Background

Although the first case of intrauterine adhesion was published in 1894 by Heinrich Fritsch, it was only after 54 years that a full description of Asherman syndrome (AS) was carried out by Israeli gynaecologist Joseph Asherman. Specifically, he identified this pathology in 29 women who showed amenorrhea with stenosis of internal cervical ostium [1]. The author speculated that such a manifestation could be a consequence of endometrium trauma. Two years later, he published another case series of intrauterine adhesions, this time involving the uterine cavity and characterized by evident filling defects during hysterography [2].

**Figure 1:**
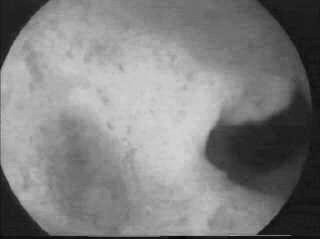
Intrauterine adhesions: hysteroscopic appearance.

Intrauterine adhesions can lead to partial or complete dysfunction of the endometrium with impairment of fertility and menstrual pattern (amenorrhea and hypomenorrhea) (Figure 1). When the adhesions are exclusively located in the lower uterine tract and functioning endometrium persists, this syndrome can also cause severe pelvic pain and retrograde menstruation.

Pregnant or early pregnant uterus seems to be more susceptible to develop uterine scarring after curettage. Nonetheless any uterine insult or trauma following even less invasive surgical procedure can lead to intrauterine adhesions development.

The impact of the AS on pregnancy is well documented with a high rate of infertility, miscarriage, poor implantation following *in vitro* fertilization and abnormal placentation [3,4].

It is important to underline that there is plenty of cases reported in literature where the presence of intrauterine adhesion (IUA) is not associated with any symptoms. Under these circumstances, some authors believe that the term of AS should be avoided [3].

Hence, AS should be defined by the presence of adhesions inside uterine cavity and/or endocervix whereby derives one or more clinical manifestations such as amenorrhea, hypomenorrhea, recurrent pregnancy loss, infertility and history of abnormal placentation.

The highest frequency of this condition was reported in Israel, Greece and South America as well as in various European countries [5]. This kind of distribution does not seem related to any specific geographic factor [5]. It is evident that the introduction of hysteroscopy in the diagnosis of intrauterine lesions has helped us to realise that IUA is much more frequent than we had previously thought [6]. Moreover, the incidence of this pathology seems to be significantly influenced by the number of abortions performed, the high incidence of genital tuberculosis in some countries and the different criteria used to detect intrauterine adhesions.

The aim of this review is to summarise the most recent evidences with regard to aetiology, diagnosis, classification and management.

## Epidemiology and aetiology

Data regarding pathophysiology of AS and IUA are still limited in literature. Electric microscopic evaluation of endometrial ghiandolar cells of women affected by severe AS revealed significant sub-cellular modifications such as ribosome lost, mitochondria swelling vascular closure and hypoxic cellular modifications [7].

Some researchers have focused their attention towards endometrial vascularity changes following endometrial trauma. An impaired vascularity of both endometrium and myometrium has been demonstrated by using pelvic angiography in patients with dense IUA [8].

In a recent prospective study involving 40 patients with AS, an high impedance of spiral artery was observed. The authors hypothesized that these phenomena could explain the reduced endometrial receptivity and regeneration in these women [9]. In addition, in patients responding to treatment, vascular endothelial growth factor (VEGF) and microvessel density are significantly increased, thus confirming that angiogenesis and revascularization may play an important role in endometrial regeneration [7].

A possible involvement of adhesion related cytokines in the pathogenesis of IUA (such as b-fibroblast growth factor, platelet derived growth factor and transforming growth factor type 1), was also recently suggested [10].

Although not clearly identified, a possible genetic factor could explain why certain patients show more frequent adhesions incidence and recurrence, or why IUA adhesions can develop even without any surgical trauma or trigger event [3,5,6].

Table 1 illustrates the most common risk factors involved in AS: it also shows that the condition is usually related to iatrogenic trauma to the endometrium. A major contribution to the identification of the main risk factors listed in Table 1 was provided by Schenker and Margalioth in a seminal study published in 1982. They evaluated 1856 cases of AS confirming that women who underwent uterine curettage were at high risk of developing intrauterine adhesions. Curettage after a miscarriage had the highest association with AS (1237 out of 1856 cases) [5]. Moreover, it was reported that when the curettage is carried out between the 2nd and the 4th postpartum week, an highest incidence of IUA is detected [6].

**Table 1:** Asherman syndrome: summary of risk factors

***Risk factors***	***Frequency***	***References***
*Miscarriage curettage*	66.7% (1237/1856)	Schenker and Margalioth 1982 [5]
*Postpartum curettage*	21.5% (400/1856)	Schenker and Margalioth 1982 [5]
*Caesarean section*	2% (38/1856)	Schenker and Margalioth 1982 [5]
*Trophoblastic disease evacuation*	0.6% (11/1856)	Schenker and Margalioth 1982 [5]
*Mullerian duct malformation*	16% (7/43)	Stillman and Asarkof 1985 [11]
*Infection (Genital tuberculosis)*	4% (74/1856)	Schenker and Margalioth 1982 [5]
*Diagnostic curettage*	1.6% (30/1856)	Schenker and Margalioth 1982 [5]
*Abdominal myomectomy*	1.3% (24/1856)	Schenker and Margalioth 1982 [5]
*Uterine artery embolization*	14 (7/51)	Mara *et al.* 2007 [12]
*Hysteroscopic surgery:*		
• metroplasty	6% (1/15)	Taskin *et al.* 2000 [13]
• myomectomy (single myoma)	31.3% (10/32)	Taskin *et al.* 2000 [13]
• myomectomy (multiple myomas)	45.5% (9/20)	Taskin *et al.* 2000 [13]
• endometrial ablation	36.4% (8/22)	Leung *et al.* 2003 [14]
*Insertion of IUD*	0.2% (3/1856)	Schenker and Margalioth 1982 [5]
*Uterine compressive sutures for post-partum haemorrhage*	18.5% (5/27)	Ibrahim *et al.* 2013 [15]

It also important to underline the fact that the number of intrauterine surgical procedure seems to be proportionally related to severity and recurrence of IUA [4].

It is not only curettage of the uterine cavity which risks AS. More recently, it has become evident that hysteroscopic surgery can also have a detrimental effect on the endometrium. According to the study by Taskin *et al.*, the hysteroscopic resection of multiple submucosal fibroids has the highest risk of IUA following hysteroscopy in a non-post gravid uterus [13].

Considering the limited number of related studies the role of the infection in the pathogenesis of AS still remains unclear [3,4].

Analysing 171 cases of caesarean sections, Polishuk *et al.* found that the 28 women who developed endometritis did not have an increased incidence of AS compared with those with no infection [16]. Based on this data, some researchers have raised doubt about the infection rule in IUA pathogenesis [17]. In addition, there is still no evidence that antibiotic therapy can exert a favourable effect after or before surgical treatment of IUA [4].

In spite of this, many authors do believe that the inflammatory pathway could play an important role on the pathogenesis of AS, resulting in the release into the intrauterine environment of factors which stimulate the formation of fibrotic tissue after endometrial trauma [18]. In conclusion, the combination of ischemia and infiammation induced by surgical trauma may constitute the main trigger for IUA development [3].

Mycobacterium tuberculosis could also involve the genital tract resulting in severe IUA [5]. In addition, genital tuberculosis seems to be associated with recurrence of IUA and poor prognosis after hysteroscopical surgery [19]. Schistosoma *sp.*, have also been implicated in the development of AS, and it has been suggested that schistosomiasis infection should be ruled out in parts of the world where it is endemic [20].

## Diagnosis

During the last 20 years, the opportunity to explore the inside of the uterus, provided by the new endoscopic procedures, has revolutionized diagnosis and management of AS. AS and IUA should be suspected in every woman presenting menstrual problems (hypomenorrhea or amenorrhea) and/or infertility with history of curettage or other intrauterine surgery. AS cannot be diagnosed by simple bimanual pelvic examination, therefore an accurate diagnosis is only possible with imaging of the uterine cavity.

Historically, hysterosalpingography (HSG) has represented the most widespread diagnostic tool. It is a cost-effective method to assess tubal patency in women who suffer from infertility. Usually, AS is characterized by filling defects described as homogeneous opacity surrounded by sharp edges [21]. In the worst cases, the uterine cavity appears completely distorted and narrowed, and ostial occlusion may also be evident. However, the information provided by an HSG is relatively crude, and it is important to bear in mind that the investigation has a high false positive rate [3].

Transvaginal ultrasound has a high compliance, and in many countries it is often used “in office” during gynaecological consultation. The ultrasounds image of AS is characterized by an echo dense pattern with difficult visualization of endometrium interrupted by one or more translucent “cyst like” areas [3]. Although, the diagnostic accuracy of ultrasound has been reported to be low [22-24], it does allow visualization of the uterine cavity when a complete obstruction of the cervix precludes HSG or hysteroscopy. Ultrasound imaging seems to be significantly influenced by ovulatory cyclical phase of menstrual cycle [25], therefore some authors suggest that the best time for the evaluation of endometrium is during luteal phase of the menstrual cycle [24].

Ultrasound control can also be useful during hysteroscopic adhesiolysis, in order to prevent uterine injury. Compared with laparoscopy, ultrasound monitoring is cheaper, with no difference in the incidence of uterine perforation [26]. In addition, some authors have reported its value to predict the outcome of surgical repair by allowing assessment of residual endometrium: if little or no endometrium is seen during transvaginal scan, the likelihood of a successful outcome is greatly decreased [27].

Data regarding the value of three-dimensional (3D) ultrasound in the detection of intrauterine adhesions are limited. Preliminary data in 2003 showed a specificity of 45% [23]. In a case series of 54 subjects with a high suspicion of AS, a significantly higher sensitivity of 3D ultrasound method was showed [28]. However, until further data becomes available, the high cost of 3D ultrasound does not justify its use in clinical practice.

The use of saline infusion during the ultrasound scan (Sonohysterography or SHG) has also been investigated. Salle *et al.* reported comparable sensitivity and specificity with the standard HSG [22]. More recently, in a retrospective study involving 149 cases with intrauterine anomalies, Acholonu *et al.* demonstrated a significant difference in general accuracy (50.3% in HSG group and 81.8% in SHG group) [29].

Another technique combining 3D ultrasound and intrauterine saline infusion (Three-dimensional sonohysterography, 3D-SHG) has recently been proposed for the diagnosis of intrauterine lesions. 3D-SHG, carried out in combination with 3D power Doppler (3-DPD), was found to have sensitivity and specificity of 91.1% and 98.8% respectively for all kinds of intrauterine lesion including synechiae [30]. Abou-Salem *et al.* confirmed these preliminary results showing comparable diagnostic efficacy with hysteroscopy [31].

Magnetic resonance imaging (MRI) can be helpful as a supplementary diagnostic tool, especially when the adhesions involve the endocervix. IUA are visualized as low signal intensity on T2 weighed-image inside the uterus [32].

Despite the above developments, hysteroscopy remains the gold standard in the assessment of AS. Table 2 illustrates the accuracy of transvaginal ultrasound, HSG and SHG compared with gold standard hysteroscopic imaging (Table 2).

**Table 2:** Diagnosis of intrauterine adhesions (Gold standard: hysteroscopy)

**Approach**	***Sensitivity (%)***	***Specificity (%)***	***PPV (%)***	***NPV (%)***	
Ultrasound	0.5	95.2	0.0	95.2	Soares *et al.* 2000 [24]
Sonohysterography	75	93.4	42.9	98.3	Soares *et al.* 2000 [24]
Hysterosalpingography	75	95.1	50	98.3	Soares *et al.* 2000 [24]

Hysteroscopy provides a real time view of the uterine cavity, allowing for a meticulous definition of the site, extent and character of any adhesions, and it is the optimum tool for assessing the endometrium. Currently, this technique can be performed in ambulatory setting with less discomfort than a blind HSG. Hysteroscopy also makes immediate treatment possible in some favourable cases [33].

## Classification

The extent of any adhesions and its impact on female reproduction should be evaluated where AS is suspected. The ideal classification system should include a comprehensive description of the adhesions which should be graded in terms of severity. Finally, it ought to provide a practical guide for clinicians to achieve optimum treatment and likely outcome.

Since Asherman original description, there have been many attempts to find the most accurate classification for IUAs. Toeff and Ballas (1978) were the first authors who tried to classify AS on the basis of hysterosalpingographic findings (Table 3) [34]. In the same year, March *et al.* introduced for the first time a hysteroscopic classification of AS (Table 4) [35]. This classification is still used for its simplicity although it is considered insufficiently prognostic [36].

**Table 3:** HSG Classification Toaff and Ballas 1978

**Classification**	**Condition**
***Type 1***	Atresia of the internal ostium, without concomitant corporal adhesions
***Type 2***	Stenosis of internal ostium, causing almost complete occlusion without concomitant corporal adhesions
***Type 3***	Multiple small adhesions in the internal ostium isthmic region
***Type 4***	Supra isthmic diaphragm causing complete separation of the main cavity form its lower segment
***Type 5***	Atresia of the internal ostium with concomitant corporeal adhesions.

**Table 4:** Classification by March 1978

**Classification**	**Condition**
***Mild***	Filmy adhesion occupying less than one-quarter of uterine cavity. Ostial areas and upper fundus minimally involved or clear.
***Moderate***	One-fourth to three fourth of cavity involved. Ostial areas and upper fundus partially involved. No agglutination of uterine walls
***Severe***	More than three fourth of cavity involved. Occlusion of both ostial area and upper fundus. Agglutination of uterine walls

Finally, the widely used classification developed on behalf of the American Fertility Society took into account the extent of the disease, menstrual pattern and the morphological feature of the adhesions. Both hysteroscopy and HSG could be used for this kind of scoring system (Table 5) [37].

**Table 5:** American fertility society classification 1988

**Classification**	**Condition**		
*Cavity involved*	<1-3	1/3 - 2/3	>2/3
	1	2	3
*Type of adhesions*	Filmy	Filmy and Dense	Dense
	1	2	3
*Menstrual pattern*	Normal	Hypo menorrhea	Amenorrhea
	0	2	4
**Prognostic classification**		HSG score	Hysteroscopy score
Stage I (Mild)	1-4		
Stage II (Moderate)	5-8		
Stage III (Severe)	9-12		

More recently, the classification published in 2000 by Nasr *et al.* illustrated an innovative way to classify AS (Table 6) [36]. This scoring system included not only the menstrual symptoms but also the obstetric history of the woman. According to this group, clinical history plays a more important role than the extent of the adhesions. The results were compared with the classifications of March and the ESH showing a good correlation in women with mild or severe disease, but not in those with moderate adhesions.

**Table 6:** Clinicohysteroscopic scoring system

***Hysteroscopic findings***		***Score***
*Isthmic fibrosis*		2
*Filmy adhesions*	More than 50% of the cavity	1
	Less than 50% of the cavity	2
*Dense adhesions*	Single band	2
	Multiple bands	4
*Tubal ostium*	Both visualized	0
	Only one visualized	2
	Both not visualized	4
*Tubular cavity* (sound less than 6)		10
***Menstrual pattern***	Normal	0
	Hypomenorrhea	4
	Amenorrhea	8
***Reproductive performance***	Good obstetrics history	0
	Recurrent pregnancy loss	2
	Infertility	4
	Mild	0-4
	Moderate	5-10
	Severe	11-22

In conclusion, there is still no clear consensus regarding the optimum classification of AS. None of proposed classification systems seems to offer a valuable reproductive prognosis, as a consequence, further studies are required [3,4].

Whether the more complex clinicohysteroscopic scoring system is any better than the others remains to be seen.

## Management and treatment of Asherman syndrome

The treatment strategy of AS could be summarized in four main steps:

1. Treatment (Dilatation and curettage, hysteroscopy, hysterotomy)

2. Re-adhesion prevention (Intrauterine device, Uterine balloon stent, Foley’s catheter, anti-adhesion barriers)

3. Restoring normal endometrium (Hormonal treatment, stem cells)

4. Post-operative assessment (Repeat surgery; diagnostic hysteroscopy; ultrasound).

## Treatment

### Hysterotomy

Few cases of AS treatment using an open-surgery approach with transfundal separation of scarring uterine walls have been mentioned: in some cases an adequate restoration of menstruation and fertility was obtained [5,38]. It has been superseded by hysteroscopic techniques, so today this strategy may be adopted only in extremely complex situation, when the hysteroscopic approach is not possible or unlikely to succeed, and only by expert surgeons [3]. The patient should be informed about the risk of the procedure, and warmed that the successful restoration of the cavity may not be obtained, not even with such an aggressive approach [3,5].

### Dilatation and curettage

Before the introduction of hysteroscopy, the blind dilation and curettage (D&C) was the treatment of choice [5]. Nevertheless blind D&C is associated with a high risk of uterine perforation as well as being a relatively poor diagnostic tool, with the result that this technique should be considered obsolete [39].

### Hysteroscopic surgery

Hysteroscopic surgery has revolutionized the treatment of intrauterine adhesion and it is the established gold standard technique. The magnification and the direct view of the adhesions allow for a precise and safe treatment. When the lesions are filmy, the tip of the hysteroscope and uterine distension may be enough to break down the adhesions [33]. Thus, in favourable cases the restoration of cavity can be obtained through “no touch” hysteroscopy in out-patient setting without general anaesthesia.

Nevertheless, the treatment of the severe and dense adhesion remains more challenging: in these cases, the cavity may be completely occluded or too narrow to allow the insertion of hysteroscopic sheath inside the cervix. Moreover, multiple procedures may be required because of post-surgical recurrence of the adhesions [3,4,6]. In these situations, it is recommended to offer a proper counselling regarding the lower rate of success and the higher risk of complications.

According to many experts, the removal of the adhesions should start form the lower part of the uterus and progress toward the upper part [3]. Any central and filmy adhesions should be separated initially in order to allow adequate distension of the uterine cavity. Dense and lateral adhesions should be treated at the end, bearing in mind the greater risk of uterine perforation and bleeding [4].

**Figure 2:**
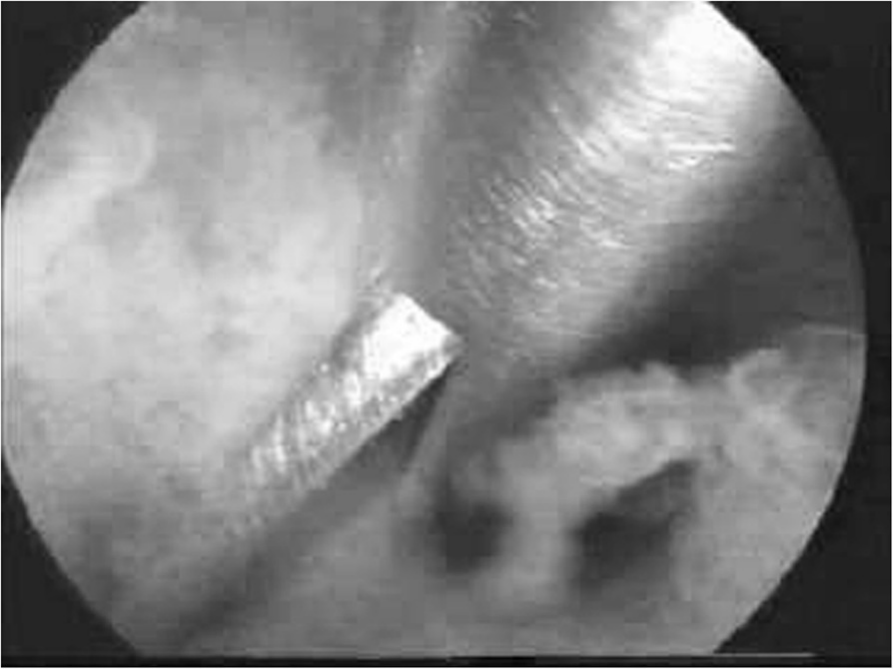
Adhesiolysis done using hysteroscopic scissors.

A wide range of mechanical or electric equipment has been adopted during hysteroscopic adhesiolysis. Even the use of a sharp needle (Touhy needle) has showed a good rate of success. Specifically, 55 patients were treated with a 16-gauge, 80-mm Touhy needle (Portex Ltd., Hythe Kent, England) introduced alongside a 5-mm hysteroscope under fluoroscopical guidance. All women regained a normal menstruation pattern but no data about fertility outcome was collected in this study [40]. A cold-knife approach is supposed to prevent thermal damage of the residual endometrium and reduce the rate of perforation during the procedure (Figure 2). The use of powered instruments (electric surgery or laser) has also proven efficient for hysteroscopic adhesiolysis [41-44]. Nevertheless the use of electric surgery is associated with potential damage to the residual endometrium [3,6].

Monopolar surgery has provided results as satisfactory as bipolar one [45]. However, one of the advantages of the latter is that the tissue effect is more focal, and the use of electrolyte-containing uterine distension media means that electrolyte changes are less likely to be clinically serious in cases of fluid overload [45].

The treatment with laser vaporization using an Nd-YAG (neodymium-doped yttrium aluminium garnet) and KTP (potassium-titanyl-phosphate) laser has also been described in the treatment of AS [46,47]. However, it is characterized by higher costs and increased uterine damages, and does not offer significant advantages over other electric equipment. Therefore its use in hysteroscopic adhesions has been increasingly abandoned [48].

The most difficult cases to treat are those with severe AS stage characterized by a complete obliteration of cavity and no apparent endometrium visible at hysteroscopy. It can be impossible to dissect the adhesions with standards hysteroscopic techniques. In these difficult circumstances, several innovative hysteroscopic strategies have been suggested in medical literature.

Mc Comb and Wagner in 1997 treated six cases with severe IUA under laparoscopic control: their technique was based on separation of uterine wall into two hemicavities by inserting a 13 French Pratt cervical dilator. Subsequently, the residual fibrotic “septum” was cut up to the fundus with hysteroscopic scissors. Regular menses was achieved in all cases, five women conceived and four of them had live births. Uterine perforation occurred in three cases, but only one case required a further hysteroscopic adhesiolysis [49].

Another innovative method was named by its creator “myometrial scoring” [50]: the technique consists in cutting six to eight incisions from the fundus of the uterus to the isthmus using a knife electrode with the aim of enlarging the uterine cavity and potentially uncovering functional endometrium. Finally, the cervix was dilated up to Hegar 12–18 to prevent cervical stenosis. Among the seven women treated with this technique, five had a normal menstrual pattern and three had conceived. Moreover, a significant reduction of the pain was achieved in two of the four symptomatic cases.

A transcervical resectoscopy after the dilatation of cervix with laminaria tent was also suggested in the treatment of severe AS [51]. Laminaria are made from dried kept stalk of the alga named “Laminaria digitata” (Shivata Medical Products Company, Nagoya, Japan). When inserted into the cervix, it slowly dilates it thanks to its hydrophilic property. The first step of the procedure consisted in the insertion into the cervix of one or two laminaria tents which were left *in situ*. After 24 hours they were replaced by three or four tents inserted up to the fundus and left *in situ* for further 24 hours. Finally, hysteroscopic resection of adhesions was carried out under laparoscopic guidance. Prophylactic antibiotics were administered during and after surgery. In addition, an intrauterine device (IUD) was placed inside the uterus and hormonal treatment with conjugated estrogens and medroxyprogesterone acetate was given. All patients enrolled in this study (*n* = 7) achieved normal menses and three became pregnant (two live birth and one miscarriage).

Hysteroscopic treatment of AS offers good results and resolves menstrual disturbance in the majority of cases [45]. Data regarding reproductive outcome came, in the majority of cases, from non randomized or prospective studies. In addition, a critical evaluation is often challenging, because of the different classification criteria and treatment strategy adopted. An overall pregnancy rate from 40% to 63% was previously described [4,44,45,52]. Fertility restoration after hysteroscopic treatment seems to be influenced by several factors such as menstrual pattern before and after the surgery, severity of adhesions and adhesions recurrence rate after treatment [53].

## Prevention of adhesion recurrence

As mentioned above, adhesions recurrence after surgery is one of the most important factors which can hinder reproductive outcome after IUA treatment. Adhesions recurrence rate is significantly higher in those cases where a severe AS is diagnosed [44,53] Several methods to prevent IUA reformations after surgery have been proposed. Nonetheless few comparative studies have been developed [54]. This could be probably due to the multitude treatment approach adopted and particularly to the lack of a unified standardized classification system for IUA diagnostic characterization.

### IUD and intrauterine adhesion

The rate of IUA reformation after surgery remains high (3.1% to 23.5%) [41,55]. These adhesions usually tend to be thin and filmy [56]. The use of IUD in order to prevent adhesion recurrence was one of the first attempts to be documented in literature [8].

It was speculated that an IUD could help physiological endometrial regeneration by separating the anterior and posterior uterine walls. Although many authors have reported good results [57,58], our data is conflicting, and there is also uncertainty about the size and the kind of the IUD to be used. Vesce *et al.* used a copper IUD with good results in 48 women with functional amenorrhea. In a short follow-up after the insertion of the IUD, a significant number of women regained a regular menses [59]. Nevertheless, some authors believe that inflammatory factors released by copper device could aggravate the endometrial injury [60]. Touguc *et al.* found no difference in adhesion reformation among women randomized to receive IUD device, estrogens treatment or no treatment after hysteroscopic septum resection [61]. The Levonorgestrel-releasing IUD should not be used for his suppressing effect on the endometrium [4]. The T-shaped IUD seems to be too tiny to guarantee a stable separation between the uterine walls [62]. With its peculiar trapezoidal shape, the Lipples loop was considered the most adequate device to prevent adhesion albeit it is no longer available in the global market [62].

### Intrauterine balloon stent

A new intrauterine stent was also described as a mechanical method to prevent adhesions recurrence [62]. It is a silicon made, triangular shape device which fits the normal triangular shape of the uterine cavity (Cook medical Inc, Bloomington, USA). In several cases treated for IUA in which the treatment protocol had included intrauterine balloon stent immediately after the procedure, good results in term of fertility outcome were achieved.

Specifically, the author reported among 1240 patients treated using intrauterine stent, pregnancy rate of 61.6% and spontaneous miscarriage rate of 15.6% [62].

No data about IUA recurrence was reported. The author recommends prophylactic broad-spectrum antibiotic until the stent remains inside uterine cavity.

In a recent cohort retrospective study of 107 patients with AS, the use of intrauterine ballon stent, compared with IUD and hyaluronic acid, resulted in significantly higher reduction of adhesions recurrence rate [63].

Although this encouraging evidence, data about its safety and efficacy seem still insufficient.

### Foley catheter

The Foley catheter was one of first mechanical devices used to separate the uterine walls preventing the recurrence of the IUA [2,35,64]. In a study involving 25 cases with moderate and severe adhesions, a fresh amnion graft over a Foley’s catheter balloon was inserted into the uterus for two weeks after hysteroscopic surgery. Although uterine perforation occurred in two cases, thus confirming the potential damage caused by this approach, a significant improvement in uterine length was found with no adhesion reformation in group with moderate adhesion [65].

The use of Foley’s catheter has also been compared with IUD as an adjunctive therapy in AS. While the IUD was removed after the third vaginal bleeding, the catheter was maintained inside the uterus for ten days. The group treated with Foley’s catheter showed higher conception rate compared with the IUD group (33.9% versus 22.5%). In addition, the 81% of women restored their normal menstrual pattern [66].

However encouraging, there are no randomized controlled trials attesting the Foley’s catheter efficacy in the prevention of IUA. The main concerns about this method are uterine perforation, ascending infection from vagina and the high discomfort.

### Hyaluronic acid and other anti-adhesion barriers

Hyaluronic acid is one of the most widespread component in human tissue and it is involved in many biological function such as mechanical support, cell migration and proliferation. In the last decades, products derived from hyaluronic acid have been adopted in gynaecologic surgery to prevent both intraperitoneal and intrauterine adhesions [67-69].

The mechanism by which these products act is not completely understood. Hyaluronic acid generates a temporary barrier between organs which mechanically obstacles adhesions formation; in addition, these products influence peritoneal tissue repair by increasing the proliferation rate of mesothelian cells [70].

Among hyaluronic based products, ferric hyaluronic acid was removed from the market due to its toxicity in 2003 [69].

Autocross-linked hyaluronic acid (Hyalobarrier©) is a new anti-adhesion barrier capable of preventing adhesion formation after gynaecological surgery (Fidia Advanced Biopolymers SRL, Padova, Italy) [71]. It is a highly viscous gel formed by the autocross-linked condensation of hyaluronic acid, and a recent systematic review confirmed that it can prevent intraperitoneal adhesion after laparoscopic myomectomy and intrauterine adhesions after hysteroscopical procedure [72].

Another anti-adhesion barrier characterized by chemically modified hyaluronic acid (sodium hyaluronate) and carboxymethylcellulose (Seprafilm©) was used for prevention of IUA (Genzyme Corporation, Cambridge, MA, USA). In a randomized controlled blind study involving 150 patients who underwent surgical evacuation or curettage after missed or incomplete abortion, the rate of IUA in the treated group was low compared with the control group [73]. There is still not enough data about long term clinical outcome, including fertility [74].

A brand new hyaluronic acid derived (alginate carboxymethylcellulose hyaluronic acid) was evaluated in a prospective randomized trial including 187 cases. Four weeks after surgery, intrauterine adhesions were significantly lower compared with carboxymethylcellulose hyaluronic acid [75].

## Restoration of normal endometrium

### Medical therapy

In order to restore basal endometrium and rebuild the normal endometrial layer inside the uterine cavity many authors have proposed hormonal treatment [33,62,76].

Many different treatments have been suggested and there is no shared consensus about the time of the administration (preoperative and/or postoperative) and the type of regimen (oestradiol or combined oestradiol and progesterone). The general idea is to encourage fast growth of any residual endometrium immediately after surgery with the dual purpose of preventing new scar formation and restoring a normal uterine environment. It is supposed that this goal can only be achieved with supraphysiological hormonal levels.

Myers *et al.* proposed a prolonged preoperative and a postoperative treatment with estrogens in 12 subjects with severe amenorrhea. All women resumed a normal menstrual pattern and six of them become pregnant [77].

March *et al.* suggested a treatment with micronized oestradiol, 2 mg twice daily for 30–60 days and medroxyprogesterone acetate 10 mg per day at last 5 days of oestrogen therapy [62].

Other authors prescribed estradiolvalerate 4 mg per day for 4 weeks and medroxyprogesterone acetate, 10 mg per day at last two week of treatment [3]. There is evidence that oestrogen-progestin treatment after curettage for post-partum haemorrhage or incomplete abortion increases endometrial thickness. Specifically, 60 women were randomized to receive estradiolvalerate 2 mg for 21 days and norgestrel 0.5 mg in the last 10 days of oestrogen treatment. 21–26 days after curettage all women underwent a transvaginal ultrasound. The endometrial thickness, width and volume were reported significantly elevated in the treated group [78].

The use of sildenafil citrate intravaginally was documented as possible pharmacological treatment to restore endometrial thickness. This drug is a type 5 specific phosphodiesterase inhibitor that enhances vasodilator effect of nitric oxide (NO) whose synthase isoforms were also found in the uterus [79]. In a prospective observational study, sildenafil citrate improved endometrial thickness in 92% of cases who presented thin endometrium (endometrial thickness <8 mm) [80]. Other encouraging results came from IVF where the combination of oestradiol and sildenafil citrate improved endometrial blood flow and endometrial thickness in 4 women with prior failed assisted reproductive cycles due to poor endometrial response [79]. There are only two case reports concerning the use of sildenafil in AS. Endometrial thickness significantly improved with treatment, and both women become pregnant after the first treatment cycle [81].

### Stem cells and endometrial regeneration

Endometrial tissue had an intrinsic capacity of regeneration. Endometrial regeneration normally occurs after menstruation and delivery. There is substantial evidence in literature that adult endometrial tissue contains epithelial progenitor cells and mesenchymal/stromal (MSC) cells [82]. These cells could be the target of a specific therapy in order to regenerate the endometrial tissue in cases of dysfunctional or atrophic endometrium. Recently, a case report of a severe AS treated with autologous stem cells isolated from the women’s own bone marrow has been described [83]. The woman had a history of infertility and hypomenorrhea following a D&C in 2005. She was treated hysteroscopically for severe intrauterine adhesions, and a T-shaped IUD was placed inside the uterus for six months. During this time, she also received therapy with combined oestrogen and progesterone (ethinylestradiol 0.05 mg from fifth to 25th day of the cycle and medroxyprogesterone acetate 10 mg from 20th to 25th day). Finally, after failure of hormonal therapy in restoring endometrium, endometrial stem cells were implanted inside the uterus after curettage on the second day of menstrual cycle. A clinical pregnancy was obtained after a heterologous embryo transfer. These pioneering discoveries could open a new scenario in the management of AS, although more evidences are mandatory.

## Post-operative assessment

Evaluation of uterine cavity after adhesiolysis is an important step in AS management. As mentioned before, complete resolution of the adhesions is not always possible with a single procedure, especially in severe stages where a high recurrence rate is documented. For instance, Valle and Sciarra reported a 50% and 21.6% of recurrence in severe and moderate AS respectively [41]. Timely recognition of any recurrence of adhesions is essential to provide the best prognosis, therefore it may be necessary to repeat surgery. For this reason, most treatment protocols include a follow-up to assess endometrial restoration after the surgery. If this is not done, there is evidence of an increased obstetric risk [62]. Although the restoration of menses is considered a good marker of success, other diagnostic investigations are fundamental for an exhaustive evaluation.

Presently, there is no clear consensus about follow up management. Usually, post-treatment assessment of the uterine cavity is recommended one-two months after the initial surgery [54]. Ultrasound, HSG and hysteroscopy are the most common follow-up methods.

Ultrasound is an accurate and cost-effective tool for measuring endometrial thickness and for the evaluation of normal endometrial development during menstrual cycle. HSG has the advantage to check tubal patency, and at the same time it can help in the resolution of thin adhesions from pressure of the liquid contrast medium. Hysteroscopy, however, remains the only method which allows an accurate estimation of adhesion recurrence and it is the most commonly used in clinical practice. Of course, it also allows further *in office* adhesiolysis.

## Conclusions

AS is a condition with a high impact on female reproduction. Even in women who conceive after AS treatment, a scrupulous surveillance should be carried out for the high risk of placental anomalies [3,5,62] and much effort should be devoted to the prevention. Considering that the highest incidence is reported after miscarriage curettage, a significant reduction of these procedure could lead to a lower incidence of AS. Specifically, the surgical removal of embryo placental retained product in condition such as incomplete or missed miscarriage should be performed conscientiously and, in favourable cases, a less invasive approach should be considered [3]. For example hysteroscopy could be an effective method for selective removal of placental retained tissue with good results in terms of pregnancy rate and IUA prevention [84]. Specifically, in a cohort study involving 95 patients, conception rate, time to conception and intrauterine adhesions was lower in the group treated hysteroscopically [84]. In addition, medical approach to miscarriage seems to significantly reduce the incidence of IUA [85]. Recent meta-analysis evaluating the safety and the effectiveness of medical treatment both for first trimester and incomplete miscarriage concluded that medical strategies are a valuable alternative to curettage [86,87].

The introduction of hysteroscopy has significantly improved the fertility outcome and the treatment success rate [3,4,62]. Nevertheless, AS recurrence rates remain high, and we must continue to look for techniques which reduce the formation of new adhesions.

IUD, uterine stent, adhesions barriers and hormonal treatment have proven efficient, yet more comparative trials are needed. A novel approach, based on endometrial stem cells and the understanding of physiopathologic mechanism involved in endometrial regeneration, could represent a worthwhile area for a future research.

## Abbreviations

3-DPD: 3D power Doppler; 3D-SHG: Three-dimensional sonohysterography; AS: Asherman syndrome; D&C: Dilation and curettage; ESH: The European Society of Hysteroscopy; HSG: Hysterosalpingography; IUA: Intrauterine adhesion; IUD: Intrauterine device; IVF: *In vitro* fertilization; KFC: Potassium-titanyl-phosphate; MRI: Magnetic resonance; MSC: Mesenchymal/stromal cells; Nd-YAG: Neodymium-doped yttrium aluminium garnet.imaging; SHG: Sonohysterography; VEGF: Vascular endothelial growth factor.

## Competing interests

The authors declare that they have no competing interests.

## Authors’ contributions

AC has reviewed the literature and prepared the manuscript. AC and ANM and gave an important contribution in revising text and preparing manuscript. ADM and GD significantly contributed to revise the article. All authors read and approved the final manuscript.
